# Associations of the metabolic score for insulin resistance and uric acid with prevalent thyroid nodules: A cross-sectional study with systolic blood pressure mediation analysis and C-TIRADS category-specific analyses

**DOI:** 10.1371/journal.pone.0358879

**Published:** 2026-09-21

**Authors:** Jiahui Ding, Xiaojin Hu, Meiling Sun, Shanshan Ge

**Affiliations:** 1 Nursing College, Shanxi Medical University, Taiyuan, China; 2 Health Management Center, The First Hospital of Shanxi Medical University, Taiyuan, China; Hong Kong Baptist University, HONG KONG

## Abstract

**Objective:**

To investigate the associations of metabolic score for insulin resistance (METS-IR) and uric acid (UA) with the odds of prevalent thyroid nodules, including nonlinear associations across C-TIRADS categories; to explore SBP-related indirect associations; and to evaluate the joint association and statistical interaction of METS-IR and UA.

**Methods:**

Among 12,256 individuals initially screened at the Health Management Center of the First Hospital of Shanxi Medical University between January 2018 and December 2021, 5,232 participants were included in the final complete-case analysis. Data were retrieved from the hospital health examination database on January 25, 2026. METS-IR and UA were categorized into quartiles. Multivariable logistic regression, restricted cubic spline (RCS) regression, exploratory mediation analysis, joint classification analysis, and interaction analyses were performed.

**Results:**

Compared with the lowest quartile, the highest quartiles of METS-IR and UA were associated with higher odds of prevalent thyroid nodules in the fully adjusted models (METS-IR: *OR*=3.928, 95% *CI*: 3.120 to 4.946; UA: *OR*=3.627, 95% *CI*: 2.839 to 4.634; both *P* < 0.001). RCS analyses showed nonlinear associations of METS-IR and UA with C-TIRADS 2–3 and C-TIRADS 4 thyroid nodules (all *P* for overall association <0.001; all *P* for nonlinearity ≤0.049). Exploratory mediation analyses yielded outcome-dependent findings: in the overall analysis, the estimated proportions mediated through SBP were 6.62% for METS-IR and 1.44% for UA; no significant indirect associations were observed for C-TIRADS 2–3 nodules, whereas only the indirect association for METS-IR was significant for C-TIRADS 4 nodules. Participants with concurrently elevated METS-IR and UA had the highest adjusted odds of prevalent thyroid nodules (*OR*, 4.562; 95% *CI*, 3.753–5.544; *P* < 0.001); however, interaction analyses indicated a submultiplicative joint association and no evidence of positive additive interaction.

**Conclusion:**

METS-IR and UA were associated with higher odds of prevalent thyroid nodules, with nonlinear associations observed across the exposure distributions. Concurrent elevations in METS-IR and UA identified a subgroup with the highest observed odds of thyroid nodules, although interaction analyses did not support positive synergy. Exploratory mediation analyses provided limited and outcome-dependent evidence of SBP-related indirect associations. The cross-sectional design precludes conclusions regarding temporal ordering or causal mediation.

## 1. Introduction

Thyroid nodules are among the most common endocrine disorders, and their detection rate has increased substantially in recent years [1]. According to the latest Chinese guidelines for the diagnosis and treatment of thyroid nodules, the prevalence of thyroid nodules in the adult is approximately 20.4%, and nearly 8%−16% of nodules are malignant [2]. Thyroid nodule occurrence has been associated with sex, age, genetic susceptibility, and iodine nutritional status [3]. Increasing evidence also suggests that metabolic abnormalities are associated with the presence and burden of thyroid nodules [4].

Insulin resistance (IR)is a central feature of metabolic syndrome [5] and has been reported to be associated with thyroid nodules after adjustment for conventional risk factors [6]. Homeostasis Model Assessment of Insulin Resistance (HOMA-IR) and Metabolic Score for Insulin Resistance (METS-IR) are commonly used surrogate markers of IR [7,8]. HOMA-IR is calculated from fasting glucose and insulin concentrations [9], whereas METS-IR incorporates fasting blood glucose (FBG), triglycerides (TG), body mass index (BMI), and high-density lipoprotein cholesterol (HDL-C) [10]. By incorporating both glycemic and lipid-related measures, METS-IR may capture metabolic abnormalities relevant to IR beyond those represented by HOMA-IR [11,12]. Previous studies have also reported that METS-IR has useful predictive performance for several metabolic outcome [13].

Elevated uric acid (UA), another common metabolic abnormality, has also been associated with a higher prevalence of thyroid nodules [14,15]. However, evidence regarding the joint association of IR-related metabolic dysfunction and UA with thyroid structural abnormalities remains limited. Both IR and elevated UA have been linked to higher blood pressure through mechanisms that may include sodium retention, activation of the renin-angiotensin-aldosterone system, endothelial dysfunction, and chronic low-grade inflammation [16–19]. Systolic blood pressure (SBP) may be particularly relevant to these associations [20]. In turn, elevated SBP has been associated with thyroid structural changes, potentially through impaired microcirculation, oxidative stress, and endothelial dysfunction [21]. Together, these observations provide a rationale for examining SBP as a potential intermediate factor in the associations of METS-IR and UA with thyroid nodules.

Therefore, this study aimed to examine the separate and joint associations of METS-IR and UA with prevalent thyroid nodules, including nonlinear associations across C-TIRADS categories. We also explored SBP-related indirect associations and assessed whether the joint association of METS-IR and UA departed from multiplicativity or additivity. By evaluating these metabolic indicators together, this study may provide additional evidence on their associations with prevalent thyroid nodules.

## 2. Materials and methods

### 2.1. Study population

A total of 5,232 individuals who underwent routine health examinations at the Health Management Center of the First Hospital of Shanxi Medical University between January 2018 and December 2021 were enrolled in this study, including 38.0% males and 62.0% females. All participants completed demographic data collection, biochemical examinations, and thyroid ultrasonography during the same health examination visit. The data used in this study were retrieved from the hospital health examination database on 25/01/2026. Based on these data, the associations between metabolic-related indicators and the prevalence of thyroid nodules were evaluated.

The inclusion criteria were as follows: (1) age ≥18 years; (2) complete demographic information; and (3) availability of complete biochemical test results and thyroid ultrasonography data. The exclusion criteria were as follows: (1) presence of thyroid-related diseases, including hyperthyroidism, hypothyroidism, or Hashimoto’s thyroiditis; (2) use of medications for thyroid diseases, such as thyroid hormone preparations or iodine-containing agents, during the study period; (3) presence of severe organic diseases, including hepatic or renal diseases, cardiovascular or cerebrovascular diseases, and malignancies; and (4) pregnancy or lactation.

**Fig 1:**
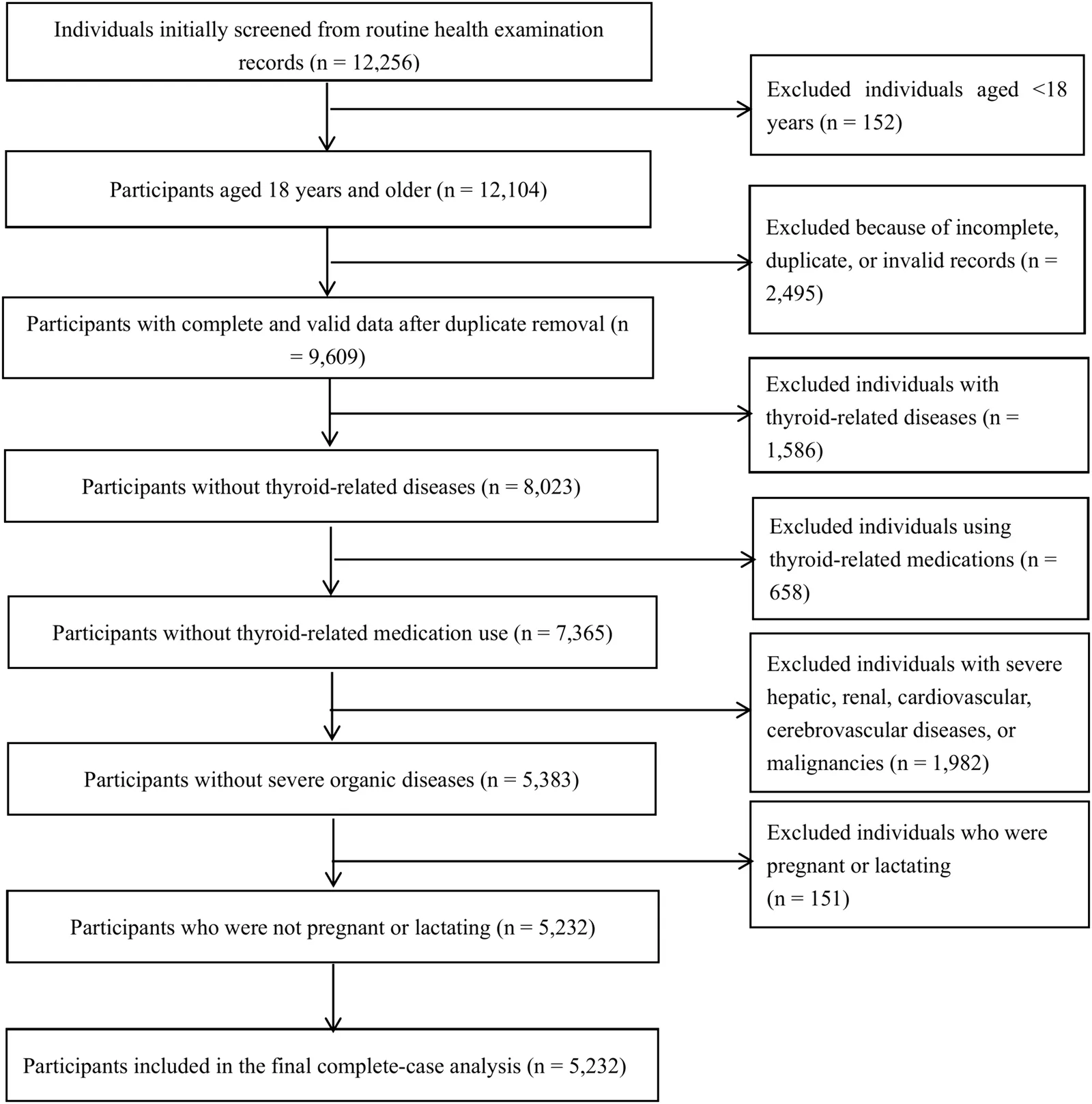
Flow diagram of participant selection.

During the study period, 12,256 individuals were initially screened. We sequentially excluded 152 individuals aged <18 years and 2,495 records because they were duplicate, incomplete, or otherwise invalid. Among the 2,495 excluded records, 43 were duplicate records, 140 had missing or invalid demographic data, 1,267 had missing or invalid biochemical data, and 1,045 had missing or invalid thyroid ultrasonography data. These exclusion categories were applied sequentially and were mutually exclusive. We subsequently excluded 1,586 individuals with thyroid-related diseases, 658 individuals using thyroid-related medications, 1,982 individuals with severe hepatic, renal, cardiovascular, or cerebrovascular diseases or malignancies, and 151 individuals who were pregnant or lactating. Finally, 5,232 participants with complete and valid records were included in the final analysis (Fig 1).

Among the 2,495 excluded records, 43 were duplicate records, 140 had missing or invalid demographic data, 1,267 had missing or invalid biochemical data, and 1,045 had missing or invalid thyroid ultrasonography data. Exclusion categories were applied sequentially and were mutually exclusive.

This study was approved by the Ethics Committee of the First Hospital of Shanxi Medical University (Approval No. KYLL-2024-045). Written informed consent was obtained from all participants prior to enrollment.

No formal sample size calculation was performed because this study included all eligible individuals with complete and valid records available during the predefined study period.

### 2.2. Data collection

The study was initiated in 2018. Basic information for all participants was collected by trained nurses using a standardized procedure, including demographic characteristics, current medical history, past medical history, medication history, and family history. Anthropometric measurements were performed using an SK-CK ultrasonic physical examination device. Participants were instructed to remove their shoes and hats and wear light clothing while standing upright on the measurement platform. Height, weight, and body mass index (BMI) were subsequently recorded.

Blood pressure was measured by trained nurses using an automated sphygmomanometer (HBP-9021; Omron Healthcare). Participants were required to rest in a seated position for at least 5 minutes before measurement. Blood pressure was measured twice, and the average value was used for analysis.

For laboratory examinations, participants were instructed to fast for at least 8 hours overnight before venous blood samples were collected in the morning. Biochemical parameters, including total cholesterol (TC), low-density lipoprotein cholesterol (LDL-C), high-density lipoprotein cholesterol (HDL-C), triglycerides (TG), fasting plasma glucose (FPG), and uric acid (UA), were analyzed using an AU5800 series automatic biochemical analyzer manufactured by Beckman Coulter.

Thyroid ultrasonography was performed by qualified sonographers. Participants were examined in the supine position with the neck fully exposed. Thyroid nodules were assessed and recorded using a color Doppler ultrasound system (EPIQ 7C; Philips Healthcare).

### 2.3. Calculation of METS-IR

The Metabolic Score for Insulin Resistance (METS-IR) was calculated using the following formula [22]


$$\begin{array}{c} METS-IR=\frac{ln(\text{2}\times FPG+TG)\times BMI}{ln(HDL-C)} \end{array}$$

where fasting plasma glucose (FPG), triglycerides (TG), and high-density lipoprotein cholesterol (HDL-C) were expressed in mg/dL, and body mass index (BMI) was expressed in kg/m^2^.

### 2.4. Diagnostic criteria

According to the 2020 Chinese Guidelines for Ultrasound Malignancy Risk Stratification of Thyroid Nodules: C-TIRADS [23], thyroid nodules were evaluated using the Chinese Thyroid Imaging Reporting and Data System (C-TIRADS). Participants classified as C-TIRADS categories 2–4 were defined as the thyroid nodule group, whereas those classified as C-TIRADS category 1 or without ultrasonographic abnormalities were assigned to the non-nodule group.

The number, size, and morphological characteristics of thyroid nodules were independently assessed and recorded by experienced radiologists. For further analysis, participants were additionally stratified according to clinically relevant risk categories. Specifically, C-TIRADS categories 2–3 were defined as the low-risk nodule subgroup, whereas category 4 was defined as the high-risk nodule subgroup. Because of the limited number of participants classified as C-TIRADS category 5, these cases were not included in the subgroup analysis.

### 2.5. Group definitions

Participants were stratified according to quartiles of METS-IR and uric acid (UA). The quartiles of METS-IR were defined as follows: Q1 (<30.26), Q2 (≥30.26 to <34.84), Q3 (≥34.84 to <40.00), and Q4 (≥40.00). The quartiles of UA were defined as follows: Q1 (<254 μmol/L), Q2 (≥254 to <311 μmol/L), Q3 (≥311 to <384 μmol/L), and Q4 (≥384 μmol/L).

In addition, participants were categorized into four groups according to the median levels of METS-IR (34.84) and UA (311 μmol/L): (1) METS-IR(-)UA(-) group, defined as METS-IR < 34.84 and UA < 311 μmol/L; (2) METS-IR(-)UA(+) group, defined as METS-IR < 34.84 and UA ≥ 311 μmol/L; (3) METS-IR(+)UA(-) group, defined as METS-IR ≥ 34.84 and UA < 311 μmol/L; and (4) METS-IR(+)UA(+) group, defined as METS-IR ≥ 34.84 and UA ≥ 311 μmol/L.

### 2.6 Quality control

Strict quality control procedures were implemented throughout the study. Participants were enrolled in accordance with predefined inclusion and exclusion criteria. Prior to participation, all individuals were fully informed of the study objectives, procedures, and contents, and written informed consent was obtained.

All medical staff involved in questionnaire administration, physical examinations, and laboratory testing received standardized training before the study. Standard operating procedures for health examinations were strictly followed during data collection. The same model of examination equipment was used for all participants, and all devices were uniformly calibrated before use. In addition, questionnaire data and examination results were independently verified and entered by two trained researchers.

## 3. Statistical analysis

All statistical analyses were performed using R software (version 4.5.1) and IBM SPSS Statistics (version 27.0). Categorical variables were summarized as frequencies and percentages and compared using the chi-square test. Non-normally distributed continuous variables were summarized as medians and interquartile ranges and compared using the Kruskal-Wallis test. Analyses were restricted to participants with complete data; no statistical imputation was performed. All tests were two-sided, and *P* < 0.05 was considered statistically significant.

Multivariable logistic regression was used to estimate the associations of METS-IR and UA with the odds of prevalent thyroid nodules. Model 1 was unadjusted, and Model 2 was adjusted for age and sex. For the METS-IR analysis, Model 3 was adjusted for age, sex, smoking history, alcohol consumption history, BUN, and UA. For the UA analysis, Model 3 was adjusted for age, sex, smoking history, alcohol consumption history, BUN, HDL-C, LDL-C, TG, TC, and FPG. SBP and DBP were not included in the primary association models because blood pressure variables may lie on the hypothesized pathways linking METS-IR and UA with thyroid nodules; SBP was examined separately as a potential mediator. Results are presented as odds ratios (ORs) with 95% confidence intervals (CIs).

For the joint classification analysis, the four groups defined in Section 2.5 were examined, with the METS-IR(-)UA(-) group as the reference. Model 1 was unadjusted, Model 2 was adjusted for age and sex, and Model 3 was additionally adjusted for smoking history, alcohol consumption history, and BUN. Multiplicative interaction was assessed by including a product term between dichotomized METS-IR and UA in the fully adjusted logistic regression model. Additive interaction was explored using the relative excess risk due to interaction (RERI), attributable proportion (AP), and synergy index (S), calculated on the OR scale. Because thyroid nodules were relatively common in this study population, the OR-based additive interaction measures were interpreted as exploratory rather than as formal risk-scale measures.

Multivariable restricted cubic spline (RCS) logistic regression was used to examine the potential nonlinear associations of METS-IR and UA with C-TIRADS 2–3 and C-TIRADS 4 nodules separately. Each RCS model used the same covariate set as the corresponding exposure-specific Model 3; that is, the METS-IR RCS models were adjusted for age, sex, smoking history, alcohol consumption history, BUN, and UA, whereas the UA RCS models were adjusted for age, sex, smoking history, alcohol consumption history, BUN, HDL-C, LDL-C, TG, TC, and FPG. Models with three to five knots were compared using the Akaike information criterion (AIC). Five knots were selected for both METS-IR models and for the UA model for C-TIRADS 2–3 nodules, whereas four knots were selected for the UA model for C-TIRADS 4 nodules. Category-specific median exposure values were used as the reference values (OR = 1): 34.58 and 33.75 for METS-IR and 309.00 and 297.00 μmol/L for UA in the C-TIRADS 2–3 and C-TIRADS 4 analyses, respectively. Overall and nonlinear associations were evaluated using Wald chi-square tests.

Mediation analyses were performed using the mediation package in R and were considered exploratory. METS-IR and UA were standardized as z scores, with the exposure contrast defined as an increase from the mean to one standard deviation above the mean. A linear regression model was fitted for SBP as the mediator, and a logistic regression model was fitted for the outcome, including the standardized exposure, SBP, and covariates from the corresponding Model 3. In the overall analysis, participants with any C-TIRADS 2–4 thyroid nodule were compared with those without thyroid nodules (n = 5,232). In category-specific analyses, participants with C-TIRADS 2–3 nodules were compared with those without thyroid nodules after excluding participants with C-TIRADS 4 nodules (n = 5,013); participants with C-TIRADS 4 nodules were similarly compared after excluding those with C-TIRADS 2–3 nodules (n = 3,950). The average causal mediation effect (ACME), average direct effect (ADE), total effect, and proportion mediated were estimated on the probability scale, with percentile-based 95% CIs derived from 5,000 nonparametric bootstrap simulations.

## 4. Results

### 4.1. Comparison of clinical characteristics between the non-thyroid nodule and thyroid nodule groups

A total of 5,232 participants were included in this study, comprising 3,731 individuals in the non-thyroid nodule group, 1,282 individuals in the C-TIRADS 2-3 group, and 219 individuals in the C-TIRADS 4 group. Among participants in the C-TIRADS 2-3 group, there were 639 males and 643 females, with females accounting for a slightly higher proportion. In the C-TIRADS 4 group, there were 123 males and 96 females, with males predominating.

Age, body mass index (BMI), systolic blood pressure (SBP), diastolic blood pressure (DBP), fasting plasma glucose (FPG), total cholesterol (TC), triglycerides (TG), uric acid (UA), and metabolic score for insulin resistance (METS-IR) progressively increased across the non-thyroid nodule group, C-TIRADS 2-3 group, and C-TIRADS 4 group, and the differences were all statistically significant (all *P*<0.001). In contrast, high-density lipoprotein cholesterol (HDL-C) levels gradually decreased across the three groups (*P*<0.001). No significant difference in alcohol consumption history was observed among the three groups (*P*>0.05). Detailed results are presented in Table 1.

**Table 1:** Comparison of baseline characteristics among participants.

Characteristics	Overall, n = 5,232	Non-thyroid nodule group,n = 3,731	Thyroid nodule group		*H/χ²*	*p* value
			C-TIRADS 2–3 group, n = 1,282	C-TIRADS 4 group, n = 219		
Age (years)	43.00 (33.00, 53.00)	41.00 (32.00, 51.00)	48.00 (35.00, 56.00)^a^	50.00 (37.00, 60.00)^ab^	175.777	<0.001
BMI(kg/m²)	23.74 (21.48, 25.99)	23.10 (20.99, 25.39)	24.98(23.08, 27.06)^a^	25.86 (23.73, 28.26)^ab^	433.815	<0.001
Sex					147.920	<0.001
Male	1,990 (38.0%)	1,228 (32.90%)	639 (49.80%)^a^	123 (56.20%)^a^		
Female	3,242 (62.0%)	2,503 (67.10%)	643 (50.20%)^a^	96 (43.80%)^a^		
Smoking history					13.659	0.001
No	4,792 (91.6%)	3,450 (92.50%)	1,149 (89.60%)^a^	193 (88.10%)^a^		
Yes	440 (8.4%)	281 (7.50%)	133 (10.40%)^a^	26 (11.90%)^a^		
Alcohol consumption history					2.196	0.334
No	4,319 (82.5%)	3,072 (82.30%)	1,072 (83.60%)	175 (79.90%)		
Yes	913 (17.5%)	659 (17.70%)	210 (16.40%)	44 (20.10%)		
SBP (mmHg)	123 (113.00, 134.00)	121.00 (112.00, 132.00)	126.00 (116.00,137.25)^a^	139.00 (127.00, 159.00)^ab^	296.289	<0.001
DBP (mmHg)	75 (68.00, 83.00)	74.00 (67.00, 82.00)	77.00 (70.00,85.00)^a^	83.00 (76.00, 92.00)^ab^	179.477	<0.001
FPG (mmol/L)	4.97 (4.66, 5.42)	4.93 (4.63, 5.35)	5.09 (4.70, 5.60)^a^	5.31 (4.82, 6.05)^ab^	112.401	<0.001
BUN (mmol/L)	4.57 (3.84, 5.38)	4.51 (3.78, 5.32)	4.77 (4.09, 5.59)^a^	4.56 (3.93, 5.38)	56.998	<0.001
TC (mmol/L)	4.92 (4.34, 5.63)	4.84 (4.29, 5.52)	5.20 (4.52, 5.85)^a^	5.22 (4.55, 5.88)^a^	100.471	<0.001
TG (mmol/L)	1.35 (0.92, 1.95)	1.24 (0.87, 1.80)	1.61 (1.16, 2.31)^a^	1.79 (1.26, 2.64)^a^	267.774	<0.001
LDL-C (mmol/L)	3.08 (2.60, 3.56)	2.98 (2.54, 3.46)	3.27 (2.76, 3.76)^a^	3.26 (2.78, 3.77)^a^	141.929	<0.001
HDL-C (mmol/L)	1.31 (1.14, 1.53)	1.35 (1.17, 1.56)	1.25 (1.09, 1.44)^a^	1.20 (1.02, 1.36)^ab^	152.908	<0.001
UA (μmol/L)	311(254, 384)	294.00 (241.00, 366.00)	346.00 (294.75, 424.00)^a^	350.00 (297.00, 438.00)^a^	373.152	<0.001
METS-IR	34.84 (30.26, 40.00)	33.37 (29.18, 38.51)	37.84 (33.44, 42.90)^a^	39.70 (35.60, 46.70)^ab^	494.427	<0.001

**C-TIRADS,** Chinese Thyroid Imaging Reporting and Data System; 1 mmHg = 0.133 kPa; **BMI,** body mass index; **SBP,** systolic blood pressure; **DBP,** diastolic blood pressure; **FPG,** fasting plasma glucose; **BUN,** blood urea nitrogen; **TC,** total cholesterol; **TG,** triglycerides; **LDL-C,** low-density lipoprotein cholesterol; **HDL-C,** high-density lipoprotein cholesterol; **UA,** uric acid; **METS-IR,** metabolic score for insulin resistance. Continuous variables were compared using the Kruskal-Wallis test, and categorical variables were compared using the chi-square test. Compared with the non-thyroid nodule group, ^a^P < 0.05; compared with the C-TIRADS 2–3 group, ^b^P < 0.05.

### 4.2. Multivariable logistic regression analysis of the associations of METS-IR and UA with thyroid nodules

After adjustment for multiple potential confounding factors in Model 3, progressively higher odds of thyroid nodules were observed across increasing quartiles of METS-IR. Using the lowest quartile (Q1) as the reference, the ORs for Q2, Q3, and Q4 were 2.235 (95% *CI*: 1.791 to 2.790; *P* < 0.001), 2.576 (95% *CI*: 2.058 to 3.226; *P* < 0.001), and 3.928 (95% *CI*: 3.120 to 4.946; *P* < 0.001), respectively. Similarly, compared with the lowest UA quartile, the ORs for thyroid nodules were 1.921 (95% *CI*: 1.544 to 2.389; *P* < 0.001) for Q2, 2.768 (95% *CI*: 2.218 to 3.454; *P* < 0.001) for Q3, and 3.627 (95% *CI*: 2.839 to 4.634; *P* < 0.001) for Q4. The detailed results are presented in Table 2.

**Table 2:** Multivariable logistic regression analysis of the associations of METS-IR and UA with thyroid nodules.

Group	Model 1		Model 2		Model 3	
	*OR*(95% *CI*)	*P*	*OR*(95% *CI*)	*P*	*OR*(95% *CI*)	*P*
METS-IR						
*Q*1	1		1		1	
*Q*2	2.770 (2.233 to 3.436)	<0.001	2.411 (1.937 to 3.001)	<0.001	2.235 (1.791 to 2.790)	<0.001
*Q*3	4.157 (3.371 to 5.127)	<0.001	3.258 (2.619 to 4.055)	<0.001	2.576 (2.058 to 3.226)	<0.001
*Q*4	7.070 (5.752 to 8.690)	<0.001	5.481 (4.394 to 6.837)	<0.001	3.928 (3.120 to 4.946)	<0.001
UA						
*Q*1	1		1		1	
*Q*2	2.322 (1.882 to 2.864)	<0.001	2.186 (1.768 to 2.703)	<0.001	1.921 (1.544 to 2.389)	<0.001
*Q*3	3.916 (3.200 to 4.793)	<0.001	3.407 (2.752 to 4.218)	<0.001	2.768 (2.218 to 3.454)	<0.001
*Q*4	5.564 (4.556 to 6.795)	<0.001	4.993 (3.960 to 6.296)	<0.001	3.627 (2.839 to 4.634)	<0.001

**METS-IR,** metabolic score for insulin resistance; **UA,** uric acid; BUN, blood urea nitrogen; **HDL-C,** high-density lipoprotein cholesterol; **LDL-C,** low-density lipoprotein cholesterol; **TG,** triglycerides; **TC,** total cholesterol; **FPG,** fasting plasma glucose. METS-IR quartiles were defined as Q1 (<30.26), Q2 (≥30.26 to <34.84), Q3 (≥34.84 to <40.00), and Q4 (≥40.00). UA quartiles were defined as Q1 (<254 μmol/L), Q2 (≥254 to <311 μmol/L), Q3 (≥311 to <384 μmol/L), and Q4 (≥384 μmol/L). For the METS-IR analysis, Model 1 was unadjusted; Model 2 was adjusted for age and sex; and Model 3 was further adjusted for smoking history, alcohol consumption history, BUN, and UA. For the UA analysis, Model 1 was unadjusted; Model 2 was adjusted for age and sex; and Model 3 was further adjusted for smoking history, alcohol consumption history, BUN, HDL-C, LDL-C, TG, TC, and FPG.

### 4.3. Nonlinear associations of METS-IR and UA with prevalent thyroid nodules across C-TIRADS categories

In the C-TIRADS-specific analyses, multivariable-adjusted restricted cubic spline regression showed significant overall associations of METS-IR and UA with the odds of prevalent C-TIRADS 2–3 and C-TIRADS 4 nodules (all *P* values for overall association < 0.001). Evidence of nonlinearity was observed for the associations of METS-IR with C-TIRADS 2–3 nodules, METS-IR with C-TIRADS 4 nodules, and UA with C-TIRADS 2–3 nodules (all *P* values for nonlinearity < 0.001). The association between UA and C-TIRADS 4 nodules also showed evidence of nonlinearity (*P* for nonlinearity = 0.049).

**Fig 2:**
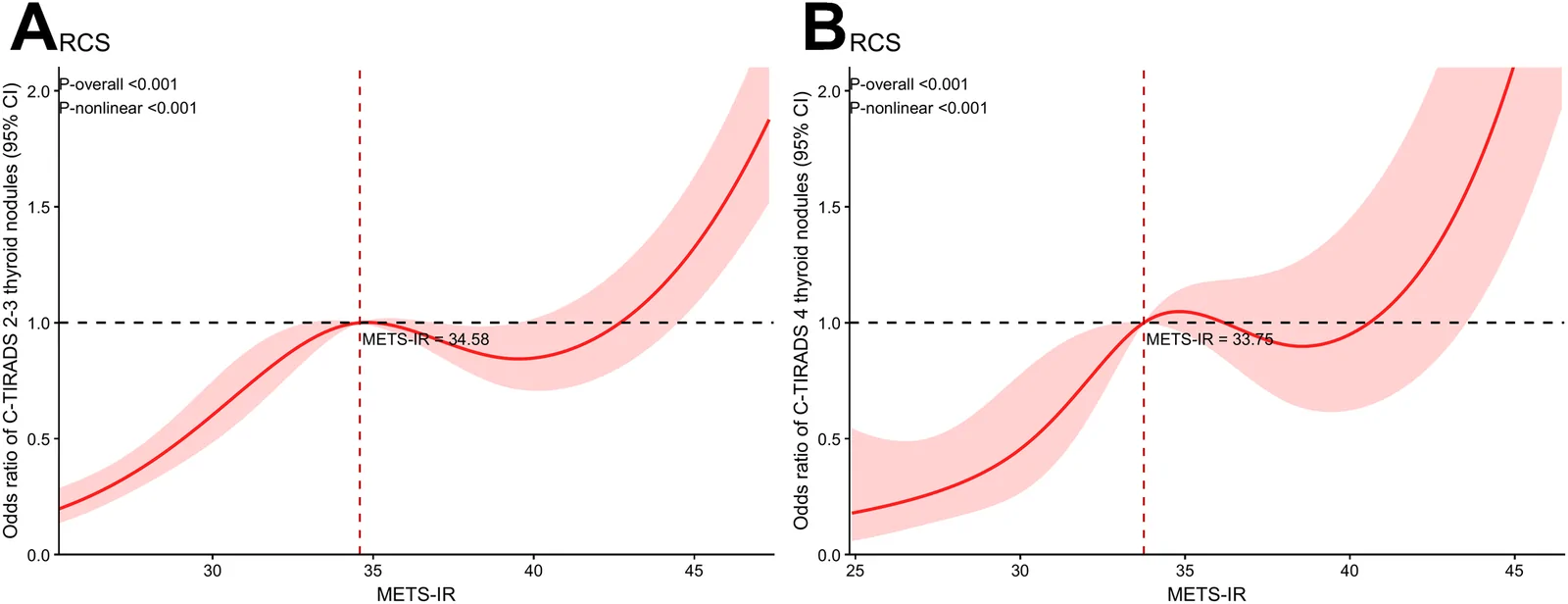
Associations of METS-IR with prevalent thyroid nodules by C-TIRADS category. **METS-IR**, metabolic score for insulin resistance; **C-TIRADS**, Chinese Thyroid Imaging Reporting and Data System; ***OR***, odds ratio; ***CI***, confidence interval. (A) Association between METS-IR and the odds of prevalent C-TIRADS 2-3 thyroid nodules. (B) Association between METS-IR and the odds of prevalent C-TIRADS 4 thyroid nodules. Both curves were estimated using restricted cubic spline regression models with five knots and were adjusted for age, sex, smoking history, alcohol consumption history, blood urea nitrogen (BUN), and serum uric acid (UA). The median METS-IR values were used as the reference values (*OR* = 1): 34.58 in panel A and 33.75 in panel B. The red solid lines and shaded areas represent the estimated *ORs* and 95% *CIs*, respectively. The black horizontal dashed line indicates an *OR* of 1, and the red vertical dashed lines indicate the reference values. *P*-overall and *P*-nonlinear represent the *P* values for the overall and nonlinear associations, respectively.

**Fig 3:**
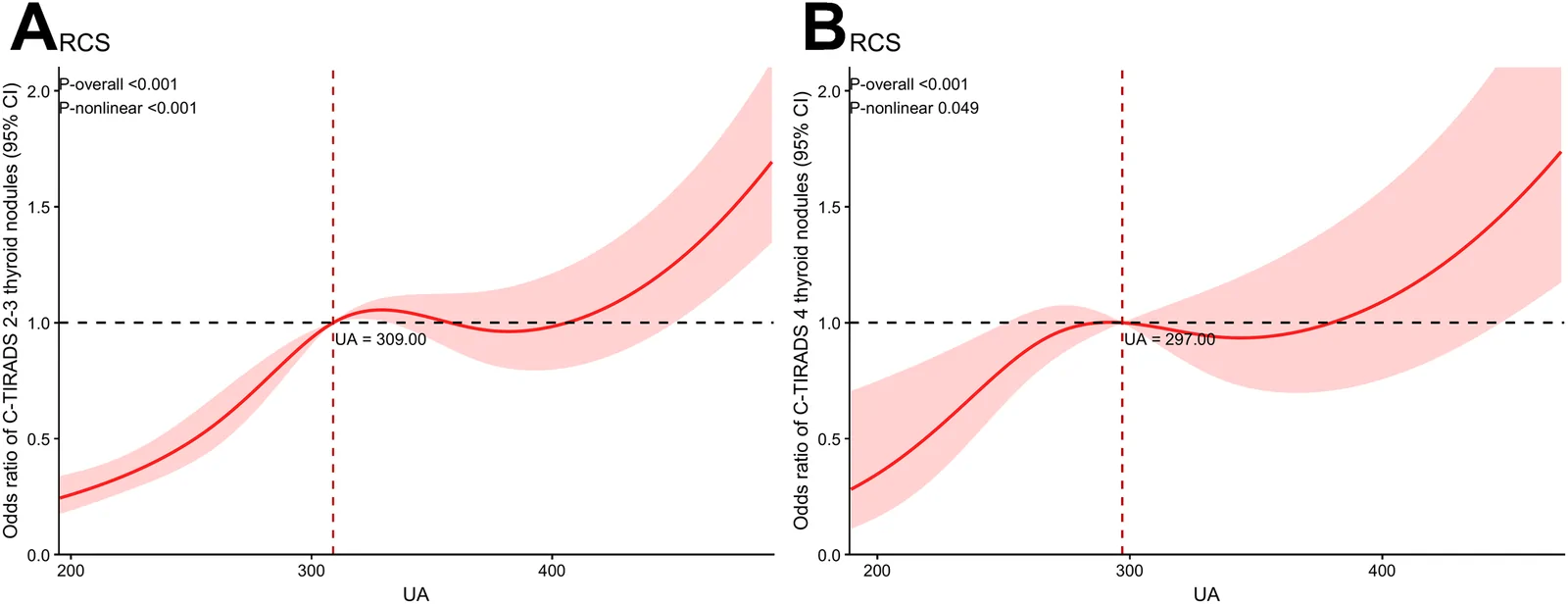
Associations of UA with prevalent thyroid nodules by C-TIRADS category. **UA**, uric acid; **C-TIRADS**, Chinese Thyroid Imaging Reporting and Data System; ***OR***, odds ratio; ***CI***, confidence interval. (A) Association between UA and the odds of prevalent C-TIRADS 2-3 thyroid nodules. (B) Association between UA and the odds of prevalent C-TIRADS 4 thyroid nodules. The restricted cubic spline regression models used five knots for C-TIRADS 2-3 nodules and four knots for C-TIRADS 4 nodules. Both models were adjusted for age, sex, smoking history, alcohol consumption history, blood urea nitrogen (BUN), high-density lipoprotein cholesterol (HDL-C), low-density lipoprotein cholesterol (LDL-C), triglycerides (TG), total cholesterol (TC), and fasting plasma glucose (FPG). The median UA values were used as the reference values (*OR* = 1): 309.00 μmol/L in panel A and 297.00 μmol/L in panel B. The red solid lines and shaded areas represent the estimated *ORs* and 95% *CIs*, respectively. The black horizontal dashed line indicates an *OR* of 1, and the red vertical dashed lines indicate the reference values. *P*-overall and *P*-nonlinear represent the *P* values for the overall and nonlinear associations, respectively.

Using the category-specific median exposure values as references (*OR* = 1), all four curves showed nonmonotonic patterns. For both METS-IR and UA, the estimated odds increased from lower exposure values toward the reference values, showed a modest mid-range flattening or decline, and then increased more markedly at the upper ends of the exposure distributions. The reference values were 34.58 and 33.75 for METS-IR and 309.00 and 297.00 μmol/L for UA in the C-TIRADS 2–3 and C-TIRADS 4 analyses, respectively. Confidence intervals widened at the extremes of the exposure distributions, particularly in the C-TIRADS 4 analyses. The associations of METS-IR and UA are shown in Figs 2 and 3, respectively.

### 4.4. Exploratory mediation analyses of SBP in the associations of METS-IR and UA with prevalent thyroid nodules

In the overall analysis, the estimated indirect associations involving SBP were statistically significant for both METS-IR (ACME, 0.0078; 95% *CI*, 0.0035 to 0.0122; *P* < 0.001) and UA (ACME, 0.0014; 95% *CI*, 0.0001 to 0.0030; *P* = 0.040). The estimated proportions mediated were 6.62% (95% *CI*, 2.91% to 10.52%) for METS-IR and 1.44% (95% CI, 0.06% to 3.02%) for UA. In the C-TIRADS 2−3 analysis, neither METS-IR nor UA showed a statistically significant indirect association involving SBP. In the C-TIRADS 4 analysis, the estimated indirect association involving SBP was statistically significant for METS-IR (ACME, 0.0153; 95% *CI*, 0.0120 to 0.0190; *P* < 0.001), with an estimated proportion mediated of 30.76% (95% *CI*, 23.89% to 40.56%). In contrast, the corresponding indirect association for UA was not statistically significant (ACME, 0.0015; 95% *CI*, −0.0004 to 0.0035; *P* = 0.134). Detailed estimates are presented in Table 3.

**Table 3:** Exploratory mediation analyses of SBP in the associations of METS-IR and UA with prevalent thyroid nodules.

Pathway	ADE, estimate (95% *CI)*	*P*	ACME, estimate (95% *CI)*	*P*	Total Effect, estimate(95% *CI)*	*P*	Proportion mediated (95% *CI)*
METS-IR → SBP → Thyroid nodules							
Overall	0.1106 (0.0943 to 0.1281)	<0.001	0.0078 (0.0035 to 0.0122)	<0.001	0.1185 (0.1025 to 0.1355)	<0.001	6.62% (2.91% to 10.52%)
C-TIRADS 2–3 group	0.1047 (0.0875 to 0.1221)	<0.001	−0.0007 (−0.0050 to 0.0034)	0.750	0.1040 (0.0879 to 0.1207)	<0.001	−0.65% (−4.79% to 3.35%)
C-TIRADS 4 group	0.0343 (0.0236 to 0.0451)	<0.001	0.0153 (0.0120 to 0.0190)	<0.001	0.0496 (0.0382 to 0.0610)	<0.001	30.76% (23.89% to 40.56%)
UA → SBP → Thyroid nodules							
Overall	0.0992 (0.0811 to 0.1181)	<0.001	0.0014 (0.0001 to 0.0030)	0.040	0.1007 (0.0825 to 0.1195)	<0.001	1.44% (0.06% to 3.02%)
C-TIRADS 2–3 group	0.0975 (0.0795 to 0.1157)	<0.001	0.0003 (−0.0001 to 0.0011)	0.232	0.0978 (0.0798 to 0.1161)	<0.001	0.32% (−0.15% to 1.15%)
C-TIRADS 4 group	0.0211 (0.0105 to 0.0327)	<0.001	0.0015 (−0.0004 to 0.0035)	0.134	0.0226 (0.0116 to 0.0343)	<0.001	6.58% (−1.97% to 17.01%)

**ADE**, average direct effect; **ACME**, average causal mediation effect; **CI**, confidence interval; **C-TIRADS**, Chinese Thyroid Imaging Reporting and Data System; **METS-IR**, metabolic score for insulin resistance; **UA**, uric acid; **SBP**, systolic blood pressure; **BUN**, blood urea nitrogen; **HDL-C**, high-density lipoprotein cholesterol; **LDL-C**, low-density lipoprotein cholesterol; **TG**, triglycerides; **TC**, total cholesterol; **FPG**, fasting plasma glucose. Estimates are reported on the probability scale for an exposure contrast from the mean to 1 SD above the mean for METS-IR or UA. Participants without thyroid nodules served as the reference group in all analyses. Models for METS-IR were adjusted for age, sex, smoking history, alcohol consumption history, BUN, and UA; models for UA were adjusted for age, sex, smoking history, alcohol consumption history, BUN, HDL-C, LDL-C, TG, TC, and FPG. Percentile-based 95% CIs were obtained from 5,000 nonparametric bootstrap resamples. Estimates were derived from separate outcome-specific models and are not directly comparable across outcome categories because the outcome definitions and prevalence levels differed.

### 4.5. Individual and joint associations of METS-IR and UA with prevalent thyroid nodules

The prevalence of thyroid nodules in the METS-IR(-)UA(-), METS-IR(-)UA(+), METS-IR(+)UA(-), and METS-IR(+)UA(+) groups was 12.29% (224/1,823), 30.52% (242/793), 31.88% (248/778), and 42.82% (787/1,838), respectively, with statistically significant differences among the four groups (*P*<0.001) (Table 4). According to C-TIRADS classification, the prevalence rates of C-TIRADS 2-3 nodules in each group were 10.92%, 28.00%, 26.35%, and 35.69%, respectively, while the corresponding prevalence rates of C-TIRADS 4 nodules were 1.37%, 2.52%, 5.53%, and 7.13%, respectively. All differences were statistically significant (*P*<0.001) (Table 4).

**Table 4:** Multivariable logistic regression analysis of the joint association of METS-IR and UA with prevalent thyroid nodules.

group	Group total (n)	Thyroid nodule			Model 1		Model 2		Model 3	
		C-TIRADS 2-3 group	C-TIRADS 4 group	Overall	*OR(*95% *CI)*	*P*	*OR(*95% *CI)*	*P*	*OR(*95% *CI)*	*P*
METS-IR(-)UA(-)	1,823	199 (10.92%)	25 (1.37%)	224 (12.29%)	1		1		1	
METS-IR(-)UA(+)	793	222 (28.00%)	20 (2.52%)	242 (30.52%)	3.135 (2.552 to 3.852)	<0.001	2.902 (2.340 to 3.600)	<0.001	2.897 (2.333 to 3.598)	<0.001
METS-IR(+)UA(-)	778	205 (26.35%)	43 (5.53%)	248 (31.88%)	3.340 (2.719 to 4.103)	<0.001	2.960 (2.402 to 3.647)	<0.001	2.946 (2.389 to 3.633)	<0.001
METS-IR(+)UA(+)	1,838	656 (35.69%)	131 (7.13%)	787 (42.82%)	5.345 (4.521 to 6.321)	<0.001	4.592 (3.781 to 5.577)	<0.001	4.562 (3.753 to 5.544)	<0.001

**C-TIRADS**, Chinese Thyroid Imaging Reporting and Data System; **METS-IR**, metabolic score for insulin resistance; **UA**, uric acid. METS-IR(-)UA(-): METS-IR < 34.84 and UA < 311 μmol/L; METS-IR(-)UA(+): METS-IR < 34.84 and UA ≥ 311 μmol/L; METS-IR(+)UA(-): METS-IR ≥ 34.84 and UA < 311 μmol/L; METS-IR(+)UA(+): METS-IR ≥ 34.84 and UA ≥ 311 μmol/L. Model 1, unadjusted; Model 2, adjusted for age and sex; Model 3, further adjusted for smoking history, alcohol consumption history, and blood urea nitrogen (BUN).

In multivariable logistic regression analysis, participants in the METS-IR(-)UA(+), METS-IR(+)UA(-), and METS-IR(+)UA(+) groups had higher odds of prevalent thyroid nodules than those in the METS-IR(-)UA(-) group after adjustment for potential confounders. The corresponding ORs were 2.897 (95% *CI*: 2.333 to 3.598; *P* < 0.001), 2.946 (95% *CI*: 2.389 to 3.633; *P* < 0.001), and 4.562 (95% *CI*: 3.753 to 5.544; *P* < 0.001), respectively. Detailed results are presented in Table 4.

In supplementary interaction analyses, the product-term estimate indicated a statistically significant departure below multiplicativity (interaction *OR*, 0.534; 95% *CI*, 0.405–0.704; *P* < 0.001). The OR-based additive interaction indices, including RERI, AP, and S, did not provide evidence of a positive additive interaction (S1 Table).

## 5. Discussion

Previous studies have reported associations of insulin resistance (IR) and uric acid (UA) with thyroid nodules, primarily using conventional regression models [24,25]. However, evidence regarding nonlinear associations and associations evaluated separately for C-TIRADS 2–3 and C-TIRADS 4 nodules remains limited. IR, elevated UA, and higher systolic blood pressure (SBP) frequently coexist in individuals with metabolic abnormalities and may be linked through shared biological processes, including chronic low-grade inflammation and oxidative stress [17,18,26]. In the present health examination population, higher METS-IR and UA were associated with higher odds of prevalent thyroid nodules. We extended previous work by examining nonlinear associations in C-TIRADS-specific analyses, exploring SBP-related indirect associations, and evaluating the joint classification and statistical interaction of METS-IR and UA.

The RCS findings indicate that the associations of METS-IR and UA with prevalent thyroid nodules were not well characterized by a single linear term. The increases observed at the upper ranges of both markers were consistent with the quartile-based findings and suggest that the associations varied across the exposure distributions. The broadly similar nonlinear patterns observed in the C-TIRADS-specific analyses further suggest that these associations were not confined to a single ultrasound-defined category. However, because the category-specific curves were estimated from separate models and no formal tests of thresholds or between-category heterogeneity were performed, the curves should not be used to infer exact inflection points or differences in association strength between C-TIRADS categories.

The marked increases in the odds of thyroid nodules at the upper ranges of METS-IR and UA may be biologically plausible. At high levels of insulin resistance, compensatory hyperinsulinemia may activate the insulin-like growth factor-1 receptor and downstream mitogen-activated protein kinase signaling, potentially promoting thyroid follicular cell proliferation. IR-related chronic low-grade inflammation may also activate nuclear factor kappa B (NF-κB) signaling and create a microenvironment that favors abnormal cellular growth [27,28]. In addition, metabolic abnormalities associated with IR may influence thyroid nodule formation through altered cellular growth and angiogenesis [29,30]. Elevated UA may further impair insulin signaling [31] and enhance pro-inflammatory cytokine responses [32], while inflammatory activity has been associated with thyroid nodule characteristics [33]. The convergence of proliferative, inflammatory, and angiogenic processes at greater levels of metabolic disturbance could therefore contribute to the pronounced upper-tail increases observed in the RCS curves. Although these mechanisms may plausibly contribute to the observed nonlinear associations, the relevant molecular and inflammatory markers were not directly measured; therefore, they should be regarded as hypotheses rather than confirmed pathways.

The mediation analyses provided limited and outcome-dependent evidence of SBP-related indirect associations. The small estimated proportions mediated in the overall models were consistent with only a limited SBP-related indirect component of the observed associations. Although the estimated proportion mediated for METS-IR was larger in the C-TIRADS 4 analysis, the category-specific estimates were derived from separate models with different analytical samples and outcome definitions and therefore cannot be interpreted as evidence of a stronger SBP-related role in this category. In addition, the proportion mediated is scale-dependent and may appear larger when the estimated total effect is relatively small. Given the cross-sectional design, these findings should be interpreted as exploratory model-based indirect associations rather than evidence of causal mediation.

The observed patterns of SBP-related indirect associations are biologically plausible. IR and compensatory hyperinsulinemia may increase renal sodium reabsorption and activate the sympathetic nervous system and renin-angiotensin-aldosterone system, thereby contributing to sodium retention, vasoconstriction, and higher SBP [17,18]. Inflammation, oxidative stress, and endothelial dysfunction may also link metabolic disturbances, including elevated UA, with higher SBP. SBP has, in turn, been associated with multinodular goiter, although the direction and underlying mechanisms of this association remain uncertain [21]. UA may additionally be associated with thyroid nodules through SBP-independent inflammatory, oxidative, or metabolic pathways. Such pathways may contribute to the heterogeneous UA-related estimates; however, the present data cannot distinguish these potential mechanisms from differences in sample composition, outcome definitions, or statistical precision. Longitudinal and mechanistic studies are needed to evaluate these possibilities.

Joint classification identified participants with concurrently elevated METS-IR and UA as having the highest adjusted odds of prevalent thyroid nodules. This finding may be useful for identifying a subgroup with a higher prevalence of thyroid nodules. However, the higher odds observed in the METS-IR(+)UA(+) group should not be interpreted as evidence of positive synergy. Supplementary interaction analyses indicated a submultiplicative joint association and no evidence of a positive OR-based additive departure. Because thyroid nodules were common in this population, the OR-derived additive interaction measures cannot be interpreted as formal risk-scale measures. Accordingly, these findings are best interpreted as supporting joint classification of METS-IR and UA rather than a positive departure from multiplicativity or additivity.

This study has several strengths, including a relatively large health examination sample, C-TIRADS-specific outcome definitions, and complementary analyses of separate, joint, and nonlinear associations. Several limitations should also be considered. First, the cross-sectional design and concurrent measurement of METS-IR, UA, SBP, and thyroid nodules precluded the establishment of temporal ordering and prevented full verification of the assumptions required for causal mediation. Accordingly, the estimated indirect and direct effects should be interpreted as exploratory model-based estimates rather than causal effects. Second, restricting the analyses to participants with complete data may have introduced selection bias, and residual confounding by unmeasured or incompletely measured factors cannot be excluded. Third, the smaller number of participants with C-TIRADS 4 nodules may have reduced the precision of the corresponding category-specific estimates. Finally, the single-center health examination population may limit the generalizability of the findings. Prospective multicenter studies with repeated measurements are needed to examine temporal relationships and potential pathways linking metabolic abnormalities, blood pressure, and thyroid nodules.

## 6. Conclusions

In conclusion, higher METS-IR and UA were associated with greater odds of prevalent thyroid nodules, with nonlinear associations observed for both C-TIRADS 2–3 and C-TIRADS 4 nodules. Concurrent elevation of METS-IR and UA identified a subgroup with the highest adjusted odds of thyroid nodules; however, supplementary interaction analyses did not support positive synergistic interaction. Exploratory mediation analyses provided limited and outcome-dependent evidence of SBP-related indirect associations. Because of the cross-sectional design, these findings cannot establish temporal ordering or causal mediation. Prospective multicenter studies with repeated measurements are needed to examine these temporal relationships and potential mechanisms.

## Supporting information

S1 Table Multiplicative and additive interaction analyses of METS-IR and UA in relation to prevalent thyroid nodules. (DOCX)

S1 File De-identified individual-level analytical dataset containing data for the 5,232 participants included in the final analysis. (XLSX)

S2 File Variable dictionary describing the definitions, units, and coding schemes for the variables included in S1 File. (XLSX)
