# Supplementary material for: Associations of the metabolic score for insulin resistance and uric acid with prevalent thyroid nodules: A cross-sectional study with systolic blood pressure mediation analysis and C-TIRADS category-specific analyses
Source: PLoS One. 2026 Sep 21;21(9):e0358879. doi: 10.1371/journal.pone.0358879 (PMC13592647; doi:10.1371/journal.pone.0358879)
Supplement: Supplementary file 1 [file pone.0358879.s001.docx]

**Table S1. Multiplicative and additive interaction analyses of METS-IR and UA in relation to prevalent thyroid nodules**

| **Interaction measure** | **Estimate** | **95% *CI*** | **Statistical interpretation** |
| --- | --- | --- | --- |
| Multiplicative interaction OR | 0.534 | 0.405 to 0.704 | *P*<0.001; |
| RERI (OR scale) | -0.282 | -1.097 to 0.534 | CI includes the null value of 0 |
| AP (OR scale) | -0.062 | -0.244 to 0.120 | CI includes the null value of 0 |
| S (OR scale) | 0.927 | 0.746 to 1.151 | CI includes the null value of 1 |

**Note:** RERI, relative excess risk due to interaction; AP, attributable proportion due to interaction; S, synergy index; OR, odds ratio; CI, confidence interval. The multiplicative interaction was estimated directly from the product term in the fully adjusted Logistic regression model. RERI, AP, and S were derived from adjusted ORs, and their 95% CIs were estimated using the delta method. Additive interaction indices were calculated on the OR scale and should be interpreted cautiously because thyroid nodules were relatively common in the study population.
